# Dissociating Markers of Senescence and Protective Ability in Memory T Cells

**DOI:** 10.1371/journal.pone.0032576

**Published:** 2012-03-02

**Authors:** Martin Prlic, Jilian A. Sacks, Michael J. Bevan

**Affiliations:** Department of Immunology and Howard Hughes Medical Institute, University of Washington, Seattle, Washington, United States of America; Saint Louis University School of Medicine, United States of America

## Abstract

No unique transcription factor or biomarker has been identified to reliably distinguish effector from memory T cells. Instead a set of surface markers including IL-7Rα and KLRG1 is commonly used to predict the potential of CD8 effector T cells to differentiate into memory cells. Similarly, these surface markers together with the tumor necrosis factor family member CD27 are frequently used to predict a memory T cell's ability to mount a recall response. Expression of these markers changes every time a memory cell is stimulated and repeated stimulation can lead to T cell senescence and loss of memory T cell responsiveness. This is a concern for prime–boost vaccine strategies which repeatedly stimulate T cells with the aim of increasing memory T cell frequency. The molecular cues that cause senescence are still unknown, but cell division history is likely to play a major role. We sought to dissect the roles of inflammation and cell division history in developing T cell senescence and their impact on the expression pattern of commonly used markers of senescence. We developed a system that allows priming of CD8 T cells with minimal inflammation and without acquisition of maximal effector function, such as granzyme expression, but a cell division history similar to priming with systemic inflammation. Memory cells derived from minimal effector T cells are fully functional upon rechallenge, have full access to non-lymphoid tissue and appear to be less senescent by phenotype upon rechallenge. However, we report here that these currently used biomarkers to measure senescence do not predict proliferative potential or protective ability, but merely reflect initial priming conditions.

## Introduction

Immunization drives proliferation and differentiation of effector CD8 T cells followed by a contraction phase after the peak of the T cell response [1], [2]. The survivors of this contraction phase go on to differentiate into long-lived memory cells, which can provide life-long protection [3]. Identifying these memory cells early on in a T cell response has been challenging due to the lack of a unique marker. Instead, the expression pattern of the molecules IL-7Rα and KLRG1 has been used to predict memory potential [4], [5]. Activated CD8 T cells that express high levels of IL-7Rα and low levels of KLRG1 are more likely to differentiate into memory cells than cells with a different expression pattern [5]. This trend holds true in several infectious disease mouse model systems including lymphocytic choriomeningitis virus (LCMV), *Listeria monocytogenes* (LM) and vesicular stomatitis virus (VSV), but not others such as influenza [6]. Following influenza infection, CD8 effector T cells express little KLRG1, but have normal contraction and memory formation characteristics [6]. KLRG1 deficient CD8 T cells are neither impaired nor enhanced in their ability to form memory following infection with LCMV or VSV [7]. Together these data demonstrate that KRLG1 expression is neither necessary nor sufficient for T cell contraction and memory formation. Similarly, IL-7Rα signaling is not sufficient to rescue activated T cells from cell death during the contraction phase [8], [9] and cells with little IL-7Rα expression on the cell surface can still differentiate into memory cells [4], [5], [10]. CD8 T cells that lack the pro-apoptotic BH3 family member Bim do not undergo apoptosis during the contraction phase, but Bim-deficient CD8 memory T cells are fully functional regardless of their IL-7Rα and KLRG1 phenotype during the priming phase suggesting that expression of these markers is not linked to memory cell function [10]. Thus, the currently used markers merely correlate with memory development in some, but not all models of infectious disease and do not play a decisive functional role in memory cell development. A similar set of markers is used to predict the proliferative potential of memory cells, typically based on KLRG1 and CD27 expression levels. Low levels of KLRG1 expression combined with high expression levels of the TNF family member CD27 have been reported to be hallmarks for fit memory cells [11]. Senescent memory cells that have lost proliferative potential have been reported to express low amounts of CD27 and high levels of KLRG1 [11], [12], [13], [14], [15]. The degree of memory cell senescence can increase with each round of memory T cell restimulation [12]. Thus, increasing the size of the memory pool by iterative stimulation comes with the potential caveat that boosting the size of the memory T cell pool could lead to T cell senescence and loss of responsiveness [12], [16], [17].

It is unclear whether cell division history dictates CD27 and KLRG1 expression. Even if it did, how could CD27 and KLRG1 regulate memory T cell fitness? CD27 has co-stimulatory properties and hence higher expression on the cell surface could be beneficial for memory cells [15]. Such a beneficial effect has been demonstrated for CD27^+^ CD8 T cells in HIV-infected patients [18]. A recent report suggests that CD27 helps to include CD8 T cells with low affinity for antigen to reach the effector and memory stage [19], while T cells with high antigen affinity are hardly dependent on CD27 expression. These data seem to rule out a comprehensive role of CD27 in regulating memory cell fitness. The connection between KLRG1 and senescence is also unclear. Given that its ligand is unknown and KLRG1 knock-out mice have no discernible phenotype [7], it is hard to hypothesize how KLRG1 expression might affect memory T cell function.

We considered that the expression pattern of CD27 and KLRG1 in memory cell populations does not reflect functional properties and is instead simply a consequence of being exposed to varying degrees of inflammation. We further speculated that the expression levels of CD27 and KLRG1 at the memory stage are not simply dictated by division history as currently postulated, but directly linked to exposure to inflammation during the primary response. Thus, we sought to establish a system that would allow us to generate effector T cells with similar division history in the context of different inflammatory environments (while keeping other variables such as antigen availability constant) and determine its impact on memory T cell formation, function and senscence. To test our hypothesis we varied the amount of inflammation by using antigen presenting activated dendritic cells (DCs) either alone or accompanied by a bystander infection with *Listeria monocytogenes* (LM) [20]. We report here that the protective ability and proliferative potential of memory T cells did not correlate with the currently used markers KLRG1 and CD27. Rather, we found that CD27 and KLRG1 expression patterns reflect the initial priming conditions of a T cell and are maintained following a tertiary and quaternary memory response. Thus, KLRG1 and CD27 expression patterns should not be used to predict memory development or fitness, but can inform initial priming conditions.

## Results

**Figure 1:**
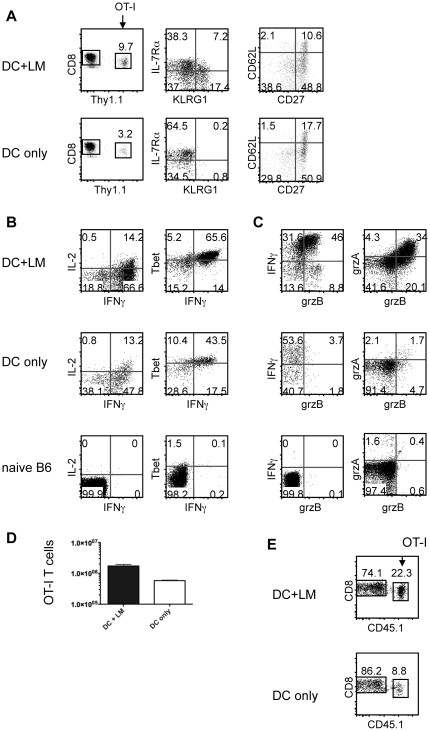
CD8 T cells do not express effector markers when inflammation is limited. C57BL/6 mice were injected intravenously (i.v.) with 1×10^6^ peptide pulsed DCs and 1×10^4^ naïve OT-I T cells (Thy1.1) with (top, DC+LM) or without (bottom, DC only) an accompanying i.v. *Listeria monocytogenes* (LM) infection. (A) OT-I T cell surface phenotype (IL-7Rα, KLRG-1, CD62L, CD27), (B) function (IL-2, IFNγ and Tbet expression) and (C) cytolytic potential (granzyme A and B) were determined 5 days post priming. Naïve polyclonal CD8 T cells are included as a baseline reference (bottom panel). (D) The number of OT-I T cells in the spleen on day 5 post priming was calculated. (E) OT-I T cell abundance in the lung was determined on day 5 post infection. The data shown are representative of up to 10 (and at least 2) independent experiments with 2–5 animals per group.

We transferred low numbers of naïve, congenically marked OT-I T cells and peptide-pulsed, LPS activated DCs into B6 hosts. Half of the animals also received a priming dose of LM. Mice from both the “DC only” group and the “DC+LM” group were sacrificed at the peak of the response (day 5) and the OT-I T cell response was analyzed. We found that OT-I T cells in both experimental groups proliferated extensively, but found consistently higher numbers of cells in the “DC+LM” group (Fig. 1A, D), which was expected based on previous studies showing that inflammation drives T cell proliferation [21], [22]. OT-I T cells that were primed in the absence of systemic inflammation (“DC only” group) did not upregulate KLRG1 (Fig. 1A). In both experimental conditions, IL-7Rα and CD62L surface expression were down-regulated, although to a lesser extent in the “DC only” group compared to the “DC+LM” group (Fig. 1A and data not shown). OT-I T cells from both experimental groups produced IFNγ, indicating that priming was successful in both cases, although cells from the “DC only” group made slightly less IFNγ than those from the “DC+LM” group (Fig. 1B). OT-I effector cells from both groups had a similarly sized population of IFNγ, IL-2 double producing cells (Fig. 1B), arguing against the possibility that the “DC only” primed OT-I T cells are early memory cells that can be generated in certain experimental conditions [23].

We found that OT-I T cells in both experimental groups expressed T-bet on day 4 (data not shown) and 5 (Fig. 1B) post activation. Expression of this transcription factor is required for optimal CD8 T cell cytolytic function and IFNγ production [24]. It has been previously shown that acquisition of cytolytic effector function and cytokine production are not necessarily directly linked events [25], [26]. OT-I T cells that were primed in an LM infected mouse expressed slightly higher levels of T-bet (Fig. 1B, right hand panels), consistent with the following phenotype: only OT-I T cells from the “DC+LM” group expressed granzyme A or B (Fig. 1C). This inflammation-dependent difference in granzyme expression was expected since inflammation is required for optimal development of CD8 effector function, while IL-2 plays a modest role [27], [28], [29], [30]. Although the level of granzyme expression appears to correlate with the number of cell divisions [31] and OT-I T cells from the “DC only” group proliferated slightly less, we wanted to address the possibility that granzyme might be expressed earlier or later. We determined granzyme expression on days 4 and 6 with the same results (data not shown). At the peak of the primary response, there were approximately 3× more OT-I effector cells in the “DC+LM” group compared to the “DC only” group (Fig. 1D) indicating that cells in the “DC+LM” group did undergo 1 to 2 more rounds of cell division assuming a similar extent of cell death between the two groups during expansion. Together, these data demonstrate that we successfully generated 2 sets of effector cells with comparable division history: maximal effectors that express high levels of T-bet, KLRG1 and granzymes (DC+LM) and minimal effectors that do not (DC only).

**Figure 2:**
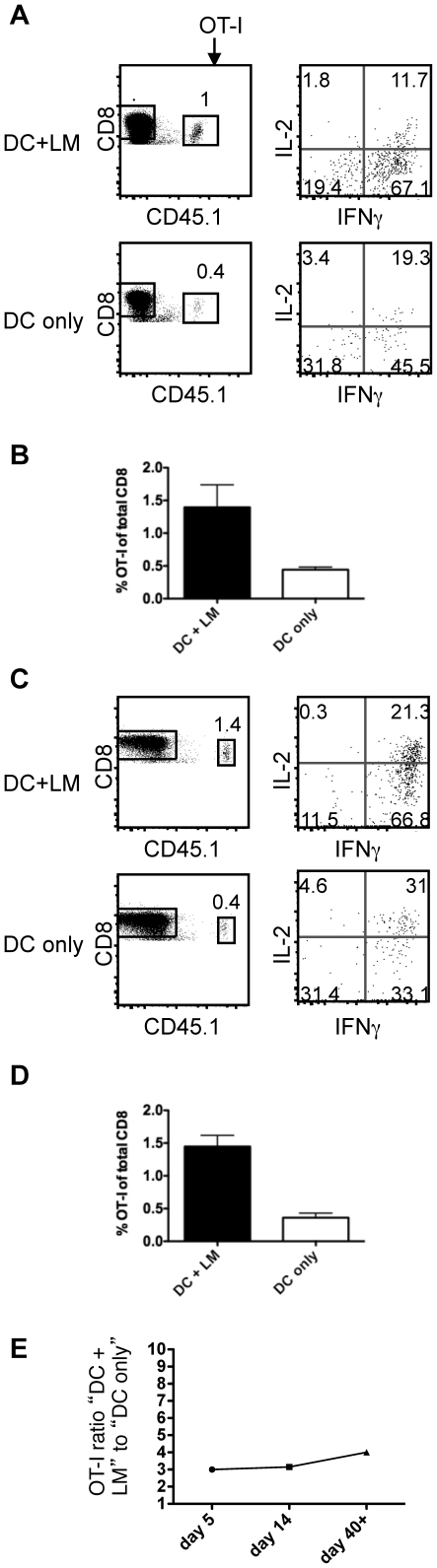
CD8 effector cells contract and are maintained as memory cells regardless of granzyme expression in the primary response. (A) OT-I T cell function (IL-2, IFNγ) and (B) abundance (displayed as percent of total CD8 T cells in spleen) were determined on day 14 post priming with peptide-pulsed DCs with (top, DC+LM) or without (bottom, DC only) a *Listeria* infection. (C) Function and (D) abundance were assessed in the spleen on day 40 or later after priming. (E) The ratio of OT-I T cells in the DC+LM versus the DC only group is plotted for the peak of the response (day 5), after the contraction phase (day 14) and during the memory phase (day 40 or later). The data are representative of at least 2 independent experiments with 2–5 animals per group.

We investigated a possible link between granzyme expression and programmed cell death during the contraction phase. We found that OT-I T cells in both groups contracted equally (Fig. 2A, B, E), showing that we generated bona fide effector cells in both groups and that acquisition of effector markers and contraction are not linked events. Next, we determined whether acquisition of cytolytic effector markers affected the functionality of the T cell pool after the contraction phase (day 14). Similar to what we had observed on day 5, OT-I T cells from the “DC+LM” group produced more IFNγ upon in vitro restimulation (Fig. 2A). Together, these data suggest that the expression patterns of KLRG-1 and T-bet during the expansion phase do not necessarily directly correlate with T cell fate in the contraction phase [32], but are rather context and model dependent.

We considered the possibility that acquisition of maximal effector function during the primary response might affect memory cell homeostasis and function over time. We found that memory cells (day 40+) from both groups were stably maintained over time (Fig. 2D, E). Thus, OT-I T cell numbers in the “DC+LM” group were about 3 to 4-fold higher compared to the “DC only” groups at all time points measured (Fig. 2E). Memory cells of both groups responded in an in vitro restimulation assay and the percentage of IFNγ producers was still reduced in the “DC only” group, while the percentage of IL-2, IFNγ double producers was similar (Fig. 2C). Taken together, these data demonstrate that acquisition of markers of effector function is not linked to memory homeostasis and minimal effectors can give rise to a fit memory population.

**Figure 3:**
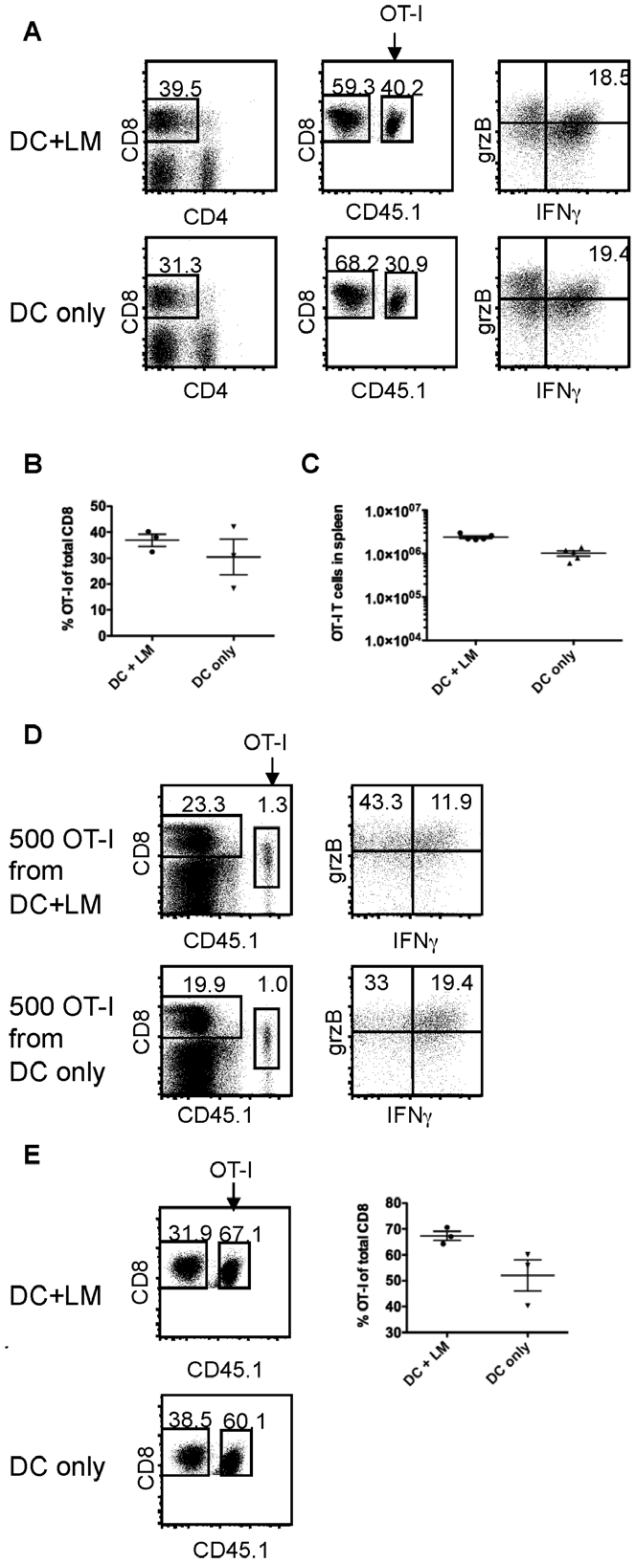
The ability of CD8 memory T cells to mount a robust recall response is independent of acquiring effector markers in the primary response. (A) 40+ days after the primary response, mice of both experimental groups were immunized with recombinant vaccinia virus expressing Ova (Vacc-OVA) [40]. The OT-I memory T cell response was analyzed 5 days later measuring T cell function (IFNγ, granzyme B) and (B) expansion. (C) 500 memory T cells (40+ days after priming) were transferred into naïve hosts and (C) expansion and (D) T cell function (IFNγ, granzyme B) were determined 7 days after infection with Vacc-OVA. (E) The OT-I memory T cell (40+ days after priming) response in the lung was analyzed 5 days after challenge with Vacc-OVA. The data shown are representative of 2 similar experiments with 3–5 animals per group.

To assess whether memory T cells generated from maximal effector and minimal effector cells are capable of mounting a robust recall response, we directly challenged the memory mice with a recombinant vaccinia strain (Vacc-OVA) on day 40 or later after the initial priming. 5 days after the rechallenge, we sacrificed the mice and analyzed T cell function and expansion (Fig. 3A, B). OT-I T cells from both experimental groups proliferated in response to the rechallenge and expressed granzyme B, as well as IFNγ (Fig. 3A). To better quantify the ability of memory cells from each group to proliferate in response to rechallenge without the limitation of having different memory precursor frequencies, we isolated memory cells from both groups and transferred 500 OT-I memory cells from “DC only” or “DC+LM” immunized mice into naïve B6 hosts and challenged with Vacc-OVA. Both memory T cell populations expanded more than 3×10^4^ fold (assuming a 10% take of the transferred cells [20]) during the secondary response, equivalent to 15+ rounds of cell division (Fig. 3C). Secondary effector cells from both experimental groups expressed similar amounts of granzyme B and IFNγ after rechallenge (Fig. 3D). Our current data show that acquisition of maximal effector function in the primary response is not necessary to generate fully functional memory cells.

In addition to determining T cell number and function in the spleen, we examined the ability of maximal and minimal effector cells to migrate to and accumulate in non-lymphoid tissue. OT-I T cells from both groups migrated to non lymphoid tissue, such as the lung during the primary response (Fig. 1E), but maximal effector OT-I T cells accumulated more, consistent with a more pronounced decrease in CD62L expression levels (Fig. 1A). Upon rechallenge, memory cells from both groups had a comparable increase in numbers of OT-I cells in the lungs (Fig. 3E), similarly to what was found in the spleen. Therefore acquisition of maximal effector function in the primary response does not affect the ability of memory cells to accumulate efficiently in non-lymphoid tissue in a recall response.

**Figure 4:**
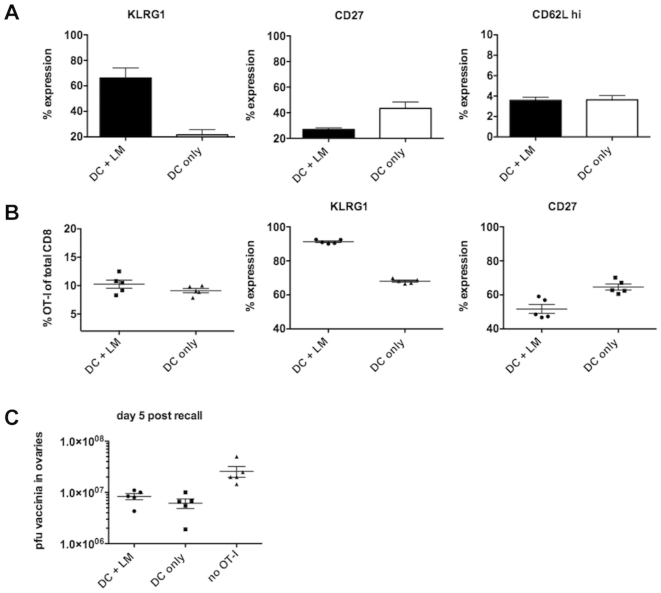
Acquisition of maximal effector function in the primary response correlates with a more senescent phenotype after a recall response, but not with proliferative potential. C57BL/6 mice were injected intravenously (i.v.) with 1×10^6^ peptide pulsed DCs and 1×10^4^ naïve OT-I T cells with (DC+LM) or without (DC only) an accompanying *Listeria monocytogenes* infection. DC only and DC+LM mice received another injection of DC only or DC+LM (2×10^5^ cfu) at least 30 days after priming, were rested for at least 30 days and then infected with VSV-OVA. These tertiary memory OT-I T cells were transferred into new B6 hosts to examine their proliferative potential and ability to clear a vaccinia infection. (A) OT-I phenotype before transfer of OT-I T cells into new B6 hosts. (B) OT-I numbers and phenotype 5 days after vaccinia infection. (C) Protective ability as measured by vaccinia pfu in the ovaries. The data shown are representative of up to 10 (and at least 2) independent experiments with 2–5 animals per group.

Finally, we created tertiary memory cells from both groups by following the priming stimulus with the same rechallenge stimulus at least 30 days later (i.e. repeat “DC only” or “DC+LM”), then boosted the animals with VSV-OVA before transferring 1×10^5^ of these memory cells into new hosts to examine their proliferative potential and ability to clear a vaccinia infection. Before transfer, the tertiary memory cells from both groups had an effector memory phenotype and had uniformly low expression of CD62L though they differed in CD27 and KLRG1 expression (Fig. 4A). We observed the same phenotypic differences (minimal effectors express less KLRG1 and more CD27) post-challenge with vaccinia (Fig. 4B), but found no difference in the ability to expand or clear virus (Fig. 4C, data not shown). Our data suggest that the initial priming conditions control the phenotype of a memory cell, including biomarkers that have been used to predict memory cell fitness (CD27, KLRG1), but do not control proliferative potential or protective ability.

## Discussion

We sought to determine the relationship between inflammation, cell division history and T cell senescence with its currently associated biomarkers KLRG1 and CD27 in memory T cells. We developed an experimental system that allowed us to regulate the extent of inflammation, while keeping other variables, such as antigen availability constant. This was achieved by transferring LPS matured, peptide pulsed DCs in the presence (high inflammation) or absence (low inflammation) of a bystander *Listeria monocytogenes* infection. Infection with LM leads to the generation of an IL-12 dominated inflammatory environment. IL-12 acts directly on IL-12 receptor expressing CD8 T cells and leads to acquisition of effector function [33], [34], [35]. Priming naïve CD8 T cells in conditions of low and high inflammation led to the generation of minimal effectors expressing low levels and maximal effectors expressing high levels of T-bet, KLRG1 and granzymes A and B, respectively. Both effector populations had a similar division history, but differed in their degree of effector function acquisition (Fig. 1B, C). Both effector populations underwent contraction to the same extent (Fig. 2A, B, E), although minimal effectors expressed uniformly low levels of KLRG1 and high levels of IL-7Rα (Fig. 1A). A similar phenotype has been previously reported for effector cells that were primed in a low inflammation setting [36]. KLRG1 expression levels directly correlated and IL-7Rα expression levels inversely correlated with the degree of inflammation present during priming (Fig. 1A). We found that both ensuing memory populations were maintained equally well (Fig. 2E) and responded equally well to a rechallenge (Fig. 3), in terms of cell expansion and cell function (Fig. 3A, C, D, E). These data argue that IL-7Rα and KLRG1 expression levels are primarily controlled by the inflammatory environment encountered during priming, but do not reliably predict cell fate (i.e. memory cell formation). The two memory populations produced similar amounts of IFNγ after rechallenge, but “DC only” memory cells tended to have a slightly higher percentage of IL-2 producing cells (Fig. 2 A, C). T cell autocrine IL-2 does not seem to play a role in T cell differentiation [37], but has been correlated with preferential effector cell survival in type I interferon driven responses [5]. Whether the observed difference is physiologically meaningful is unclear and potential consequences on cell fate and function will require further investigation. Although functionally similar, the ensuing two memory populations differed phenotypically whereby expression levels of CD27 and KLRG1 reflected initial priming conditions even in tertiary memory populations (Fig. 4A) that had been generated by rechallenge with a viral pathogen (VSV-OVA). Memory cells that arose from minimal effectors expressed less KLRG1 and more CD27 than memory cells derived from effectors primed under inflammatory conditions. We wanted to determine whether CD27 and KLRG1 expression could accurately predict the proliferative and functional potential of memory cells. A positive correlation would have suggested that the initial priming conditions could potentially affect long-term memory fitness. Instead, we found that the original phenotypic differences in the two effector populations were still present in memory populations even 5 days after rechallenging tertiary memory cells (i.e. quaternary memory): memory cells derived from minimal effectors expressed less KLRG1 and more CD27 (Fig. 4B), but expression levels of KLRG1 and CD27 had no impact on the quality of the memory response as measured by the ability to proliferate and clear a viral infection (Fig. 4B, C). Together, these data suggest that KLRG1 and CD27 expression on memory cells can reflect the original priming conditions, but do not predict memory cell fitness.

Clearly, acquisition of maximal effector function in the primary response is not required to generate CD8 memory and to gain maximal effector function after rechallenge, but how does this insight impact our current approach to vaccination? One concern about repeatedly stimulating T cells is that these cells can lose their memory characteristics and are pushed towards an effector phenotype characterized by poor recall ability and loss of replicative potential with each round of restimulation [12], [16], [38]. We found that memory cells were skewed towards an effector memory phenotype and uniformly CD62L low prior to a quaternary challenge (Fig. 4A), but still capable of robust expansion when rechallenged (Fig. 4B), indicating that a loss of proliferative potential is not characteristic of all prime boost strategies. In summary, the protective ability and proliferative potential of a memory T cell can be sustained even after several rounds of prime-boost stimulation and memory cell fitness does not correlate with currently used phenotypic markers.

Our data provide evidence that the initial priming conditions are imprinted in memory T cells, even after several rounds of restimulation. Thus, the initial priming conditions dictate aspects of the memory phenotype, including the currently used markers CD27 and KLRG1 of senescence. Our data show that these markers are not suitable for predicting T cell fitness and a novel set of biomarkers, ideally with known functional properties and ligands [7], will be required to accurately predict T cell protective and proliferative potential.

## Materials and Methods

### Ethics Statement

This study was approved by and all experiments were done in accordance with the University of Washington Institutional Animal Care and Use Committee (protocol number 2015-01). The UW School of Medicine is fully accredited by the American Association for Accreditation of Laboratory Animal care. An Animal Welfare Assurance is on file with OPRR-NIH.

### Mice

C57BL/6 and RAG KO mice were obtained from The Jackson Laboratory (Bar Harbor, ME) and housed in specific pathogen-free conditions in the animal facilities at the University of Washington (Seattle, WA). OT-I TCR transgenic mice congenic for CD45.1 or Thy1.1 were bred and maintained in the same facilities.

### Adoptive transfer and cell sorting

Naïve CD44^low^ OT-I T cells were isolated from lymph nodes as previously described [20]. A total number of 1×10^4^ naïve OT-I T cells per recipient was transferred. Transfer of memory cells: splenic CD8 T cells were enriched by depleting CD4^+^, CD19^+^ and I-Ab^+^ cells in a magnetic column (Miltenyi) and a population of CD8 T cells containing 500 or 1×10^5^ memory OT-I T cells was injected into naïve recipients.

### Dendritic cell isolation

Dendritic cells (DC) were expanded in B6 mice with a Flt-3L secreting mouse melanoma cell line. CD11c^+^ cells were purified, LPS (1 µg/ml) and peptide (1 µg/ml) pulsed in vitro prior transfer as previously described [20].

### Infections

*Listeria monocytogenes* was grown as previously described [20]. For primary infections, mice were injected i.v. with 2×10^3^ cfu LM, previously infected mice received 2×10^5^ cfu LM. In some experiments memory cells were boosted with a recombinant VSV strain expressing the SIINFEKL epitope [39]. After transfer of secondary or tertiary memory CD8 T cells, new recipient mice were infected with 2×10^6^ pfu of a recombinant vaccinia strain expressing the SIINFEKL epitope [40] and were sacrificed 5 to 7 days later. Vaccinia pfu was determined in ovaries of RAG mice 5 days post T cell transfer and infection.

### Flow Cytometry

Recipient mice were sacrificed at the time points indicated. For intracellular staining, cells were prepared with the Cytofix/Cytoperm kit in the presence of brefeldin A (BD) and incubated with or without 100 nM SIINFEKL peptide for 4–5 hours in complete RP10. Cells were analyzed using a FACSCanto (BD) and analyzed using FlowJo (TreeStar) software
